# Short Term Effects of Particle Exposure on Hospital Admissions in the Mid-Atlantic States: A Population Estimate

**DOI:** 10.1371/journal.pone.0088578

**Published:** 2014-02-07

**Authors:** Itai Kloog, Francesco Nordio, Antonella Zanobetti, Brent A. Coull, Petros Koutrakis, Joel D. Schwartz

**Affiliations:** 1 Department of Geography and Environmental Development, Ben-Gurion University of the Negev, Beer Sheva, Israel; 2 Department of Environmental Health - Exposure, Epidemiology and Risk Program, Harvard School of Public Health, Boston, Massachusetts, United States of America; 3 Department of Biostatistics, Harvard School of Public Health, Boston, Massachusetts, United States of America; German Diabetes Center, Leibniz Center for Diabetes Research at Heinrich Heine University Duesseldorf, Germany

## Abstract

**Background:**

Many studies report significant associations between PM_2.5_ (particulate matter <2.5 micrometers) and hospital admissions. These studies mostly rely on a limited number of monitors which introduces exposure error, and excludes rural and suburban populations from locations where monitors are not available, reducing generalizability and potentially creating selection bias.

**Methods:**

Using prediction models developed by our group, daily PM2.5 exposure was estimated across the Mid-Atlantic (Washington D.C., and the states of Delaware, Maryland, New Jersey, Pennsylvania, Virginia, New York and West Virginia). We then investigated the short-term effects of PM_2.5_ exposures on emergency hospital admissions of the elderly in the Mid-Atlantic region.We performed case-crossover analysis for each admission type, matching on day of the week, month and year and defined the hazard period as lag01 (a moving average of day of admission exposure and previous day exposure).

**Results:**

We observed associations between short-term exposure to PM_2.5_ and hospitalization for all outcomes examined. For example, for every 10-µg/m^3^ increase in short-term PM _2.5_ there was a 2.2% increase in respiratory diseases admissions (95% CI = 1.9 to 2.6), and a 0.78% increase in cardiovascular disease (CVD) admission rate (95% CI = 0.5 to 1.0). We found differences in risk for CVD admissions between people living in rural and urban areas. For every10-µg/m^3^ increase in PM _2.5_ exposure in the ‘rural’ group there was a 1.0% increase (95% CI = 0.6 to 1.5), while for the ‘urban’ group the increase was 0.7% (95% CI = 0.4 to 1.0).

**Conclusions:**

Our findings showed that PM_2.5_ exposure was associated with hospital admissions for all respiratory, cardio vascular disease, stroke, ischemic heart disease and chronic obstructive pulmonary disease admissions. In addition, we demonstrate that our AOD (Aerosol Optical Depth) based exposure models can be successfully applied to epidemiological studies investigating the health effects of short-term exposures to PM_2.5._

## Introduction

Fine Particulate Matter (PM_2.5_- particles with an aerodynamic diameter≤2.5 µm) is a complex mixture of particles primarily composed of sulfate (SO4), nitrates (NO3),ammonium (NH4), elemental carbon (EC), organic compounds (OC), and various metals [1]. Air pollution and particularly, PM_2.5_ has consistently been associated with increased hospital admissions in cities throughout the United States and the world [2]–[5]. Exposure to airborne PM_2.5_ can increase hospital admissions for various causes [6]–[8], [5], [9], [10]. The causes associated with short-term PM_2.5_ exposure include *inter alia* admissions for: all respiratory causes [11], [12], chronic obstructive pulmonary disease –COPD [13]–[15], cardiovascular disease-CVD [16], [17], stroke [18], ischemic heart disease (IHD) [19], myocardial infarction (MI) [3] and diabetes [2].

The majority of these epidemiologic studies have used available PM_2.5_ monitors located within their study domain. Since PM_2.5_ concentrations vary spatially within the study domain this introduces exposure error and likely produces a combination of downward bias in the effect estimates and wider confidence intervals due to a mixture of classical and Berkson error [20].

A key study conducted by Zanobetti and colleagues [6] looked at the association between two-day mean PM_2.5_ and emergency hospital admissions in 26 US communities. They estimated the association between daily PM_2.5_ and emergency hospital admissions for CVD, MI, congestive heart failure (CHF), respiratory disease, and diabetes in 26 US communities, for the years 2000–2003. Using meta-regression, Zanobetti and colleagues examined whether this association was modified by season and community specific PM_2.5_ composition, after controlling for seasonal temperature as a surrogate for ventilation. They found that for every 10 µg/m^3^ increase in 2-day averaged PM_2.5_ exposure there was an increase of 1.9% (95% CI: 1.3 to 2.4) in CVD, 2.2% (95% CI: 1.1 to 3.4) in MI, 1.8% (95% CI: 1.3 to 2.5) in CHF, 2.7% (95% CI: 1.3 to 4.2) in diabetes, and 2.1% (95% CI: 1.2 to 2.9) in respiratory admissions.

Belleudi and colleagues [21] investigated the impact of PM_2.5_ and ultrafine particles (particulate matter with a diameter of less than 100 nanometres in diameter) on emergency hospital admissions for cardiac and respiratory diseases. More specifically, they evaluated the effect of PM exposures on emergency hospital admissions in Rome between 2001–2005 on acute coronary syndrome, heart failure, lower respiratory tract infections, and COPD. PM data were collected daily at one central fixed monitor. Data were analyzed with a case-crossover analysis using a time-stratified approach. Belleudi and colleagues reported an immediate impact of same-day to exposure to PM_2.5_ on hospitalizations for acute coronary syndrome of 2.3% (95% CI: 0.5% to 4.2%) and an increase of 2.4% for heart failure (95% CI: 0.3% to 4.5%). The effect on lower respiratory tract infections showed an increase of 2.8% (95% CI: 0.5% to 5.2%) for a 2-day lag.

Most previous studies have been limited by the lack of high spatial and temporal resolution of daily exposure data. For many of the previous US studies PM_2.5_ data was available only for one in three or one in six days. In addition, all these studies were limited to populations living close to monitoring stations and thus did not include individuals living in suburban and rural areas where no monitoring stations were available. Further, geographic differences in the daily variability of exposure were usually not captured.

We have recently presented a new method of assessing temporally- and spatially-resolved PM_2.5_ exposures for epidemiological studies, and applied it to data from the Mid-Atlantic region of the U.S. [22]. A recent paper published by our group, which is an extension of our previous published models [23], allows us to estimate spatially resolved PM_2.5_ on a daily basis throughout the Mid-Atlantic states. In this paper, we use our model generated predictions to study the association between short-term PM_2.5_ exposure and emergency hospital admissions among elderly (aged 65 and older) included in the Medicare program across the Mid-Atlantic region. Medicare is a national social insurance program, administered by the U.S. federal government since 1966,that guarantees access to health insurance for Americans aged 65 and older. We take advantage of our geographic resolution to examine the effect of space dependent modifiers such as poverty or education, as well as effect estimate differences between more and less urbanized settings. In addition, our study investigates the entire population of a region, rather than selected locations near monitoring sites as commonly done in previous studies.

## Methods

This study was approved by the institutional review boards of the Harvard School of Public Health. The US Medicare data is previously collected administrative data and does not require individual patient consent. All Hospital admittance records were anonym zed.

### Study Domain

**Figure 1:**
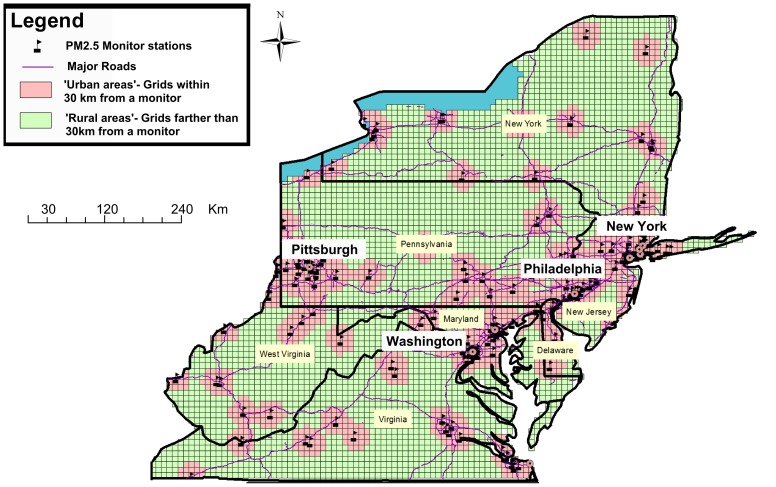
Map of the study area showing the MEDICARE population within and outside 20_2.5_ monitor.

The presented study’s spatial domain included the Mid-Atlantic region comprising of Washington D.C., and the states of Delaware, Maryland, New Jersey, Pennsylvania, Virginia, New York and West Virginia (Figure 1).

The data cover an area of 495,486 km^2^ with a population of 57,999,568. The average size of population in the Mid-Atlantic zip codes for the general population is 9095 and 1246 for people 65 and over. The median zip code population is 2818 for the general population and 420 for people 65 and over. Around 36.2% of the Medicare population in our analysis (4,531,059) live within 30 km of a monitor station (more ‘urban’ areas) while 63.8% (7,998,329) live farther then 30 km from a monitoring station (more ‘rural’ areas). In general the ‘rural’ population is poorer with lower median income levels of $37,327 compared to $49,928 in the ‘urban’ areas and lower levels of education, with only 13.9% of people holding bachelor level degrees compared to 23.0% in the ‘urban’ areas. Figure 1 shows the population areas that are within and further than 30 km of a monitor.

### Data

#### Exposure data

**Figure 2:**
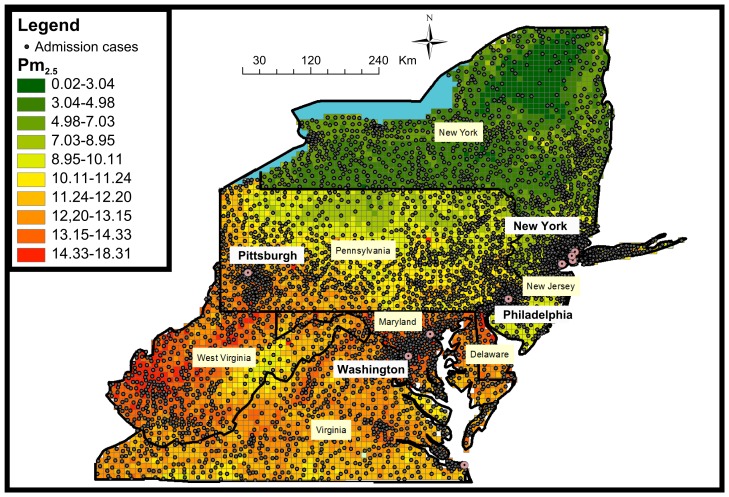
Map of the study area showing the residential location of admission cases by zipcode (the centroids of the zipcodes) juxtaposed over a sample PM_2.5_ 10×10 km pollution grid for 01/06/2001.

PM_2.5_ exposures for the years 2000–2006 were assessed using our recently developed prediction models [22] that incorporate satellite AOD (Aerosol Optical Depth) data. The Mid-Atlantic exposure dataset encompasses daily PM_2.5_ predictions at a 10×10 km spatial resolution across the study area (Figure 2) during the entire study period.

We used ground PM_2.5_ measurements from 161 monitoring sites from the EPA (Environmental Protection Agency) and IMPROVE (Interagency Monitoring of Protected Visual Environments) monitoring networks and AOD data from the Moderate Resolution Imaging Spectroradiometer (MODIS) satellites. We also incorporated land use regression (elevation, distance to major roads, percent of open space, point emissions and area emissions) and meteorological variables (temperature, wind speed, relative humidity and visibility). In stage 1 of the model, we calibrate the AOD grid-level observations to the PM_2.5_ monitoring data collected within 10 km of an AOD reading. The first stage of the model consists of a mixed model for observed PM_2.5_ (containing both fixed and day-specific random effects for the intercept), the AOD slopes, and the temperature slopes. We then incorporate the additional spatial, temporal (daily), and spatio-temporal covariates as predictors in the PM_2.5_ model. To accommodate the fact that daily AOD data missingness is not random, the first stage model incorporates inverse probability weighting (IPW) to potentially prevent bias in the regression coefficient estimates and thus in the resulting estimations. To accommodate the fact that the PM-AOD calibration factors can vary spatially between large regions, we divided the Mid Atlantic area into regions. The intercept, AOD, and temperature random effects in the model are nested within regions of the study. In stage 2 of the model, we estimate PM_2.5_ concentrations in grid cells without monitors but with available AOD measurements using the stage 1 fit. Finally, in stage 3 of the model, we estimated daily PM_2.5_ concentration levels for all grid cells in the study domain for days when AOD data were unavailable. Using the PM_2.5_ predictions obtained from the first stage of the model as the response, we fit a model containing a smooth function of latitude and longitude (of the grid cell centroid) and a random intercept for each cell. This is similar to universal kriging, extended to include the mean of the PM_2.5_ monitors on that day (the average PM_2.5_ concentrations measured at all the available PM_2.5_ monitors in the region on each day) and random cell-specific slope. To allow for temporal variations in the spatial correlation, a separate spatial surface was fit for each two-month period of each year. Using this method provides additional information about the concentration in the missing grid cells that simple kriging would not. To validate our model, we repeatedly divided the data randomly into 90% and 10% splits. Predictions for the held-out 10% of the data were made from the model fit of the remaining 90% of the data. This “out of sample” process was repeated ten times, and cross-validated (CV) R^2^ values were computed. Even for location-day combinations without AOD data our model performance was still excellent (mean out-of-sample R^2^ = 0.81). Both the stage 1 and stage 3 models yielded very small predictions errors (RMSPE - Root of the mean squared prediction errors) −1.1 µg/m3 and 1.4 µg/m3 respectively, indicating a strong model performance. Further, CV results revealed no bias in the predicted concentrations (Slope of observed vs. predicted = 0.97–1.01).

PM_2.5_ exposure estimates were generated by our prediction models. These PM_2.5_ daily predictions were matched to zip codes using ArcGIS and SAS based on spatial location and date. For more detailed information on the prediction model please refer to Kloog et al [22].

#### Hospital Admittance data

Individual hospital admittance records were obtained from MEDICARE and cover hospitalization for all residents of age 65 and older, for all available years (2000–2006). We defined cases as those with an emergency admission and a primary discharge diagnosis using ICD-9 (International classification of diseases, ninth revision) for all respiratory (ICD 9 460–519), CVD (ICD 9 390–459), IHD (ICD 9 410–414),COPD (ICD 9 490–496) and stroke (ICD 9 434) related admissions. These records included information such as age, sex, date of admission, race/ethnicity, and zip code of residence.

We choose broader areas for the leading admission causes (CVD and all respiratory, at a cost of loss of specificity) since one would expect the broader admission data to produce less noisy estimates for two reasons. First, the counts are higher and therefore there is more power to examine CVD admissions. Secondly, studies of misdiagnosis in hospital administrative records show that the broader the categories, the less the amount of misclassification. We also were interested in specifically looking at the COPD and stroke admissions sub category associations with PM_2.5_ to compare to previous studies [21]. The US Medicare data is previously collected administrative data and does not require individual patient consent.

#### Covariates

Temperature data were obtained through the National Climatic Data Center (NCDC) [24]. Only continuous operating stations with daily data running from 2000–2006 were used. For meteorological variables zip codes were matched to the closest weather station. All *Socioeconomic* variables were obtained through the US Census Bureau from the 2000 social, economic and housing characteristics datasets [25]. S*ocioeconomic* variables used included the following zip code level information: Percent of minorities, age, education (people with no high school education) and median income.

### Statistical Methods

Zip code-specific admissions were matched with our exposure estimates for each 10×10 km grid cell. We used a case-crossover analysis approach, which was developed as a variant of the case-control design to study the effects of transient exposures on acute events [26]. This design samples only cases and compares each subject’s exposure experience in a time period just before a case-defining event with that subject’s exposure at other times. Because there is perfect matching on all measured or unmeasured subject characteristics that do not vary over time, there can be no confounding by those characteristics. If in addition, the control days are chosen to be close to the event day, slowly varying subject characteristics are also controlled by matching. We used this time stratified approach in our analysis. We matched on day of the week and defined the relevant exposure time window as the mean exposure of the day of and day before the patient’s hospital admission.

The data were analyzed using conditional logistic regression (PROC PHREG, release 8.2; SAS Institute, Cary, NC). Temperature with the same moving average as PM_2.5_ was included in the model as a potential confounder.

Case-crossover analyses lend themselves to the analysis of effect modification and thus we looked at several interactions. Specifically, in the largest admission group (CVD) we investigated whether the subject residence within 30 km of a monitor or farther than 30 km from monitor modified the PM_2.5_ association with admissions. In addition we examined interactions between exposure and both income level (low vs high income groups) and gender.

To investigate the robustness of our results, various sensitivity analyses were run on the CVD and all respiratory admissions. Specifically, we analyzed other averaging periods: lag02 (a moving average of day of admittance exposure and 2-days of previous exposure) and lag 0 (day of admittance exposure) vs. lag01 (a moving average of day of admittance exposure and previous day exposure). We also matched control days to be days within the same month and year, with the same temperature (rather than the same day of week), to control for temperature by matching rather than modeling [27]. In that case, we used dummy variables to control for day of the week.

## Results

Descriptive statistics are presented in Table 1. The majority of people included in our analyses who were admitted to hospitals were white (85%–88% across all admission causes) while the average age was 77.5–79.6 years. In total, more than 2 million admissions were included in the study.

**Table 1:** Descriptive statistics for hospital admissions by type of admission across the Mid-Atlantic for the years 2000–2006.

Characteristic	All Respiratory	CVD	Stroke	COPD	IHD
	No. (%)	No. (%)	No. (%)	No. (%)	No. (%)
**Sex**					
Male	744,761 (43.20)	1,382,379 (45.64)	294,113 (41.40)	176,314 (42.30)	618,518 (51.62)
Female	979,135 (56.80)	1,646,478 (54.36)	416,228 (58.60)	240,464 (57.70)	579,681 (48.38)
***Race***					
White	1,492,579 (86.58)	2,615,049 (86.34)	603,419 (84.95)	367,836 (88.26)	1,058,448 (88.34)
Black	178,822 (10.37)	321,631 (10.62)	85,524 (12.04)	38,505 (9.24)	100,385 (8.38)
other	52,495 (3.05)	92,177 (3.04)	21,398 (3.01)	10,437 (2.50)	39,366 (3.29)
**Age (years)**	79.56	78.61	79.43	77.49	77.21

Table 2 contains a summary of the predicted exposures for both the acute PM exposure (2 day moving average- lag01 for overall area and rural/urban areas) and temperature across all grid cells in the analysis.

**Table 2:** Descriptive statistics for short-term PM _2.5_ exposure and temperature in the Mid-Atlantic for 2000–2006.

Covariate	Mean	Min	Max	Median	SD	Range	IQR	Q1	Q3	Days of data available
**Lag01 PM _2.5_** **(µg/m^3^)**	11.92	0.01	95.85	10.78	5.68	96.56	6.73	7.92	14.65	2557
**Lag01 PM _2.5_** **(µg/m^3^) - Rural**	11.53	0.01	95.17	10.45	5.51	95.88	6.53	7.65	14.18	2557
**Lag01 PM _2.5_** **(µg/m^3^)- Urban**	12.81	0.01	95.85	11.58	5.97	96.07	7.09	8.60	16.68	2557
**Temperature** **(°F)**	49.08	−15.10	87.90	50.20	18.40	103.00	28.95	35.90	64.85	2557

Note: Q1 and Q3 are quartiles.

Table 3 presents the estimated percent increase, and associated 95% confidence intervals, in hospital admissions for a 10 µg/m^3^ increase in PM_2.5_ by cause of admission. For example, for all respiratory admissions, we found a 2.2 percent increase in admissions (95% CI = 1.9 to 2. 6). For CVD admissions, we found a 0.8 percent increase (95% CI = 0.5 to 1.0). COPD, IHD and stroke all showed similar increase in admission rates (see Table 3).

**Table 3:** Estimated percent increase in hospital admissions for a 10 µg/m^3^ increase in short-term PM_2.5_ by cause of admission.

All Respiratory	CVD	Stroke		COPD	IHD
% increase	% increase	% increase		% increase	% increase
2.23 (1.91–2.56)	0.78 (0.54–1.01)	0.11 (−0.36–0.59)		1.83(1.18–2.48)	0.99(0.62–1.37)
**CVD Interactions**	**% increase and p-value of the interaction term**				
‘rural’ group	1.04 (0.56 to 1.51)		p = 0.21		
‘urban’ group	0.7 (0.44 to 0.96)		p = 0.21		
low income group	1.10 (0.62 to 1.58)		p = 0.13		
high income group	0.69 (0.43 to 0.95)		p = 0.13		
Males	0.83 (0.50 to 1.17)		p = 0.37		
Females	0.73 (0.42 to 1.04)		p = 0.37		

We found differences (*albeit* not significant based on the p-value of the interaction term) in the PM_2.5_ associations with CVD between people living closer to monitor areas (‘urban’ group) and farther away (‘rural’ group), between the income level groups (high and low) and small differences between the genders (see Table 3).

The results from the sensitivity analysis were consistent with the primary analysis. Both for CVD and all respiratory admissions, when using lag0 or lag02 PM_2.5_,we found significant associations that were smaller in lag0 (0.7%, 95% CI = 0.4 to 0.9 and 1.89%, 95% CI 1.5 to 2.0 respectively) and very similar in lag02 compared to the main lag01 analysis (0.8%, 95% CI 0.6 to 1.0 and 2.1%, 95% CI 1.8 to 2.5 respectively). The results from matching control days to be days with the same temperature resulted in very similar results to the main analysis. For CVD, we found a 1.0 percent increase in admission rate (95% CI = 0.6 to 1.3).

## Discussion

In this paper we examine associations between PM_2.5_ exposures generated by our novel prediction model and increased hospital admissions in an elderly population (aged 65 and older) in the Mid-Atlantic States. These associations were positive for all respiratory, CVD, COPD,IHD and stroke admission causes tested. We also found differences in the PM_2.5_ associations between people living closer to monitor areas and farther away, between the income level groups and small differences between the genders. The associations observed in our analysis are broadly consistent with the associations observed in many recent case-crossover analysis examining associations between short-term PM_2.5_ exposures and hospital admissions [7], [21].

Figures 1 and 2 clearly show the large areas that commonly have been left out of most previous analysis and now can be included in future studies. This is especially important in areas like the Mid-Atlantic region where a large percentage of the population indeed lives in areas that are very far from monitoring sites. These more ‘rural’ population tends to poorer and have lower levels of education compared to the more ‘urban’ areas. This ability now to look at all the Mid-Atlantic region gives enough power to take a look at subset of admission diagnosis (COPD, IHD, Stroke) and not just the large admissions groups (CVD, All respiratory), which was one of the primary aims of this research. Moreover, we found some indication of differences in the association in those areas. Specifically, the increase in CVD admissions associated with PM_2.5_ was larger in the less densely populated areas, which were not previously included in most analyses. This is of great importance to risk assessment studies since it may provide some evidence about the differential toxicity of fine particles between ‘urban’ and ‘rural’ Mid-Atlantic elderly populations. In less urban areas secondary particles, for example sulfate, represent a larger fraction of fine particle mass compared to those in urban areas. Acidic sulfate compounds such as ammonium bisulfate and sulfuric acid which are present in fine particles measured in the Mid-Atlantic region [28] have been shown to increase the levels of soluble transition metals in particles, which generate reactive oxygen species (ROS) [29]. The harmful effects of ROS on the cell are most often damage of DNA, oxidations of polyunsaturated fatty acids in lipids (lipid peroxidation), oxidations of amino acids in proteins and oxidatively inactivate specific enzymes by oxidation of co-factors [30] which may lead to *inter alia* tumor promotion [31]. Alternatively, these differences in effect estimates may be related to differences in exposure factors (e.g., individuals living in the less urban areas spend more time outdoors) or differences in health factors (e.g., lifestyles and access to close by health care- in rural areas health care centers may be far away from the place of residence and this can influence the number of admissions, and the timing of the admission from the onset of symptoms). This represents an important extension of previous Medicare analyses, since we now have estimates for suburban, small town, and rural populations.

To the best of our knowledge, this is the first time that all the population in the Mid-Atlantic area, and not just those close to monitors, have been included in an analysis of the association between PM_2.5_ exposures and hospital admissions. The use of our spatiotemporal model reduces exposure misclassification that may exists in, for example, time series studies that use a single exposure metric for daily exposure in an entire metropolitan area. Such error is a mixture of classical exposure error, which likely biases the effect estimates downward, and Berkson error, which increases the confidence interval [20]. Our results show much tighter confidence intervals compared to the classic time series analysis, indicating that our method could potentially reduce measurement error. For example for CVD, the width of our 95% confidence intervals of 0.5–1.0 is much smaller than the comparable intervals reported by Zanobetti et al. [6], who reported 95% confidence intervals of 1.3–2.4. All respiratory causes show the same pattern (95% CI: 1.9 to 2.6, compared to 95% CI: 1.2 to 2.9).

There are a few limitations in the presented study. The spatial resolution (zip codes for the Medicare data) are not individual addresses, but those are not available because of privacy concerns. In addition, the use of 10×10 km for the satellite data could be improved as well. However, as satellite remote sensing evolves and progresses, higher spatial resolution data (such as 1×1 km) should become available soon and will further reduce exposure error [32]. Such finer resolution should enable us to assess more precise estimated daily individual exposure as they relate to different location such as residence and work place for datasets where individual addresses are available.

## Conclusion

In conclusion, our findings indicate that hospital admissions for all respiratory, CVD, IHD, COPD and stroke were associated with PM_2.5_ exposures. In addition, we have demonstrated that our AOD-based exposure models can be successfully applied to epidemiologic studies investigating the health effects of short-term exposures to PM_2.5_. This is because these models make it possible to estimate spatially-resolved PM_2.5_ exposures for specific zip codes. In addition, they can be used to assess exposures for large regions which allows for the inclusion of both urban and rural populations. This provides more generalizable results for risk assessment.
